# Pulsatile stretch as a novel modulator of amyloid precursor protein processing and associated inflammatory markers in human cerebral endothelial cells

**DOI:** 10.1038/s41598-018-20117-6

**Published:** 2018-01-26

**Authors:** Sumudu V. S. Gangoda, Bhargava Avadhanam, Nurul F. Jufri, Eun Hwa Sohn, Mark Butlin, Vivek Gupta, Roger Chung, Alberto P. Avolio

**Affiliations:** 1 Department of Biomedical Sciences, Faculty of Medicine and Health Sciences, Macquarie University, Sydney, Australia; 2 Programme of Biomedical Science, Faculty of Health Sciences, Universiti Kebangsaan Malaysia, 50300 Kuala Lumpur, Malaysia; 3 Department of Herbal Medicine Resources, Kangwon National University, Samcheok, 25949 Republic of Korea; 4 Department of Clinical Medicine, Faculty of Medicine and Health Sciences, Macquarie University, Sydney, Australia

## Abstract

Amyloid β (Aβ) deposition is a hallmark of Alzheimer’s disease (AD). Vascular modifications, including altered brain endothelial cell function and structural viability of the blood-brain barrier due to vascular pulsatility, are implicated in AD pathology. Pulsatility of phenomena in the cerebral vasculature are often not considered in *in vitro* models of the blood-brain barrier. We demonstrate, for the first time, that pulsatile stretch of brain vascular endothelial cells modulates amyloid precursor protein (APP) expression and the APP processing enzyme, β-secretase 1, eventuating increased-Aβ generation and secretion. Concurrent modulation of intercellular adhesion molecule 1 and endothelial nitric oxide synthase (eNOS) signaling (expression and phosphorylation of eNOS) in response to pulsatile stretch indicates parallel activation of endothelial inflammatory pathways. These findings mechanistically support vascular pulsatility contributing towards cerebral Aβ levels.

## Introduction

Alzheimer’s disease (AD) is the most common form of dementia. Post-mortem brain tissue examination reveals amyloid plaques, which are considered to play an important role in the pathophysiology of AD^1^. The main constituent of amyloid plaques, amyloid β (Aβ) peptides, are derived from amyloid precursor protein (APP), a transmembrane protein expressed in various cell types including endothelial cells (ECs)^1^.

Although AD is conventionally classified as a neurodegenerative disease, emerging evidence indicates that dysregulation of vascular factors is a common feature of disease progression^2–4^. Of the multitude of vascular factors implicated in AD, involvement of vascular pulsatility, endothelial dysfunction and inflammatory changes in ECs are far from clear^2,3,5^. However, there is evidence in the literature indicating that these factors are concomitant with AD^2–4^.

Higher pulsatility index and pulse pressure, indicative of increased large artery stiffness and reduced vessel compliance, are associated with AD, lower memory scores, increased amyloid burden and cerebral microvascular damage^3,4^. Vascular stiffness is also related to endothelial dysfunction where nitric oxide (NO)-mediated-endothelium-dependent vasodilation, which is facilitated by the enzyme, endothelial NO synthase (eNOS), is diminished. Inhibition or deficiency of eNOS was found to be associated with higher APP protein expression and secretion of Aβ, while Aβ was found to mediate vascular dysfunction by hampering eNOS-mediated NO release^6–8^.

Additionally, vascular stiffness could lead to increased vascular inflammation, which is also known to be associated with AD^5,9^. Elevated expression and localisation of inflammatory markers such as intercellular cell adhesion molecule-1 (ICAM-1) surrounding AD-plaques have been demonstrated^5^. Together, these data corroborate the findings that high pulsatility, endothelial dysfunction and vascular inflammation are independently associated with AD^5,6,8^.

These factors are also present in the milieu of hypertension, which is a major risk factor for AD^4^. Although direct correlations of antihypertensive therapy and dementia remains to be clarified, there is compelling evidence indicating that hypertension poses additive effects on cognitive impairment and that anti-hypertensive therapy could alleviate the deleterious cognitive effects^10,11^. Increased systolic pressure and decreased diastolic pressure have been shown to be positively correlated with increased amyloid burden, risk of AD incidence, reduced cognitive function and increased gray matter atrophy^4,11,12^. Notably, increased pulse pressure, vascular stiffness and hypertension could impose increased mechanical stretch on microvessel walls. The consequence of this chronic stress placed upon cerebral microvascular ECs is currently unknown.

The present study investigated whether increasing pulsatile stretch magnitude as a mechanistic stimulus in a cerebral microvascular EC culture model, could modulate processes such as expression and processing of APP, Aβ secretion, endothelial dysfunction and inflammation, all of which are contributors to AD-pathology.

## Results

### Pulsatile stretch modulates cerebral endothelial expression of APP

**Figure 1:**
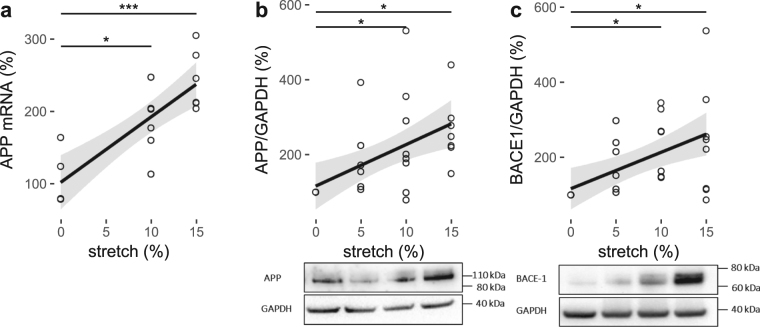
The effect of pulsatile stretch on APP and β-secretase 1 (BACE-1) in HCMECs. % control mRNA expression (**a**) and densitometric analysis of % control band intensity relative to GAPDH and the representative western blots of protein expression of (**b**) APP and (**c**) BACE-1 expression indicating amyloidogenic processing of APP at different magnitudes of pulsatile (1 Hz) stretch of HCMECs over 18 hours. ***P < 0.001, *P < 0.05. n = 5–10, analyzed using One-way ANOVA with post-hoc Tukey-corrected multiple comparison tests. Solid line shows the linear regression for the data and the shaded region the 95% confidence interval of the regression (regression statistics in Table 1).

After HCMECs were stretched for 18 hours at 0%, 5%, 10% or 15% stretch magnitudes, the qPCR analysis of APP mRNA expression was significantly higher than the static control (105 ± 17%) at 10% (184 ± 19%, P < 0.05) and 15% (243 ± 17%, P < 0.001; Fig. 1a) stretch. Linear regression analysis showed a significant linear relationship between stretch and APP mRNA expression (Table 1).

**Table 1:** Regression statistics (linear model) showing changes with respect to increasing cyclic (1 Hz) stretch magnitude from 0 to 15%, as displayed in Figs 1–5.

	slope	intercept	R^2^	p
APP mRNA (%)	9.04	102	0.67	**<0.0001**
APP/GAPDH (%)	11.08	116	0.30	**0.0022**
BACE1/GAPDH (%)	9.70	117	0.29	**0.0023**
Aβ40 (pg/ml)	−0.024	6.9	0.05	0.1998
Aβ42 (pg/ml)	0.028	0.6	0.21	**0.0041**
Aβ42/Aβ40 ratio	0.0043	0.08	0.21	**0.0038**
eNOS mRNA (%)	1.09	102	0.04	0.4542
eNOS/GAPDH (%)	9.11	108	0.21	**0.0055**
peNOS/GAPDH (%)	−4.96	98	0.72	**<0.0001**
ICAM/GAPDH (%)	2.42	92	0.17	**0.0109**
LDH activity (units/ml)	11.79	739	0.04	0.2458

Consistent with the overall increase in mRNA expression in response to stretch, the western blot results of protein expression of APP also showed the same trend (Fig. 1b, Table 1) under the same stretch conditions. APP expression was also significantly higher at 10% (240 ± 52%, P < 0.05) and 15% (265 ± 34%, P < 0.05) of pulsatile stretch magnitudes compared to the static control (100%; Fig. 1b).

### Pulsatile stretch results in amyloidogenic processing of APP and Aβ42 secretion in cerebral ECs

As for the APP expression, the protein expression of APP processing enzyme, β-secretase 1 (BACE-1), was measured in response to 18 hours of stretch at 0%, 5%, 10% or 15% stretch magnitudes using western blotting. BACE-1 was also increased at 10% (239 ± 32%, P < 0.05) and 15% (242 ± 52%, P < 0.05) stretch magnitudes (Fig. 1c), consistent with the expression of APP with linear regression showing significant increase with stretch magnitude (Fig. 1c, Table 1).

**Figure 2:**
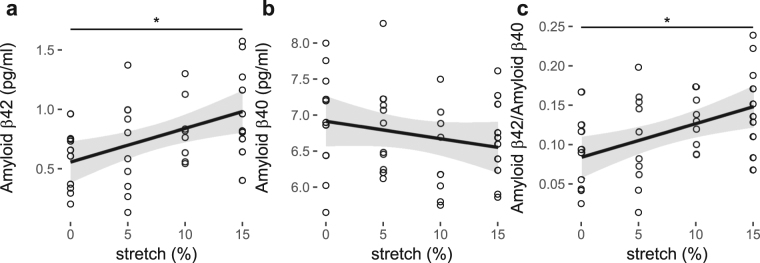
Amyloid β (**a**) 40 and (**b**) 42 secretion and (**c**) the ratio of the two for increasing magnitude of pulsatile (1 Hz) stretch of HCMECs over 18 hours. *P < 0.05. n = 5–10, analyzed using One-way ANOVA with post-hoc Tukey-corrected multiple comparison tests. Solid line shows the linear regression for the data and the shaded region the 95% confidence interval of the regression (regression statistics in Table 1).

The secreted Aβ42 levels in the supernatants from ECs that were stretched at 0, 5, 10 or 15% stretch, showed a significant positive linear relationship with increasing magnitude of pulsatile stretch (Fig. 2a, Table 1). Aβ40 levels did not change with stretch (Fig. 2b, Table 1) resulting in an increase in the Aβ42/Aβ40 ratio with stretch magnitude (Fig. 2c, Table 1).

### Pulsatile stretch modifies the expression of eNOS in cerebral ECs

**Figure 3:**
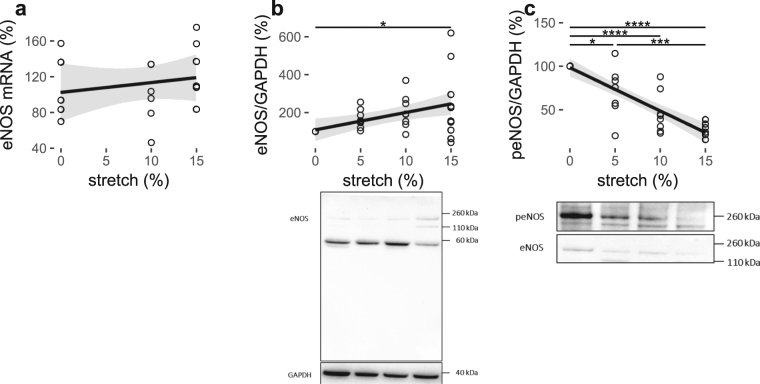
Pulsatile stretch modulates eNOS expression and phosphorylation in HCMECs. % Control mRNA expression (**a**) densitometric analysis of % control band intensity relative to GAPDH and the representative western blots of protein expression (**b**) and phosphorylation at S1177 (**c**) of eNOS in response to pulsatile (1 Hz) stretch of HCMECs over 18 hours. ****P < 0.0001, ***P < 0.001 *P < 0.05. n = 5–10, analyzed using One-way ANOVA with post-hoc Tukey-corrected multiple comparison tests. Solid line shows the linear regression for the data and the shaded region the 95% confidence interval of the regression (regression statistics in Table 1).

The mRNA and protein expression of eNOS was also quantified after HCMECs had been subjected to 0%, 5%, 10% or 15% stretch for 18 hours using qPCR and western blotting respectively. There were no differences in eNOS mRNA expression at individual levels of stretch magnitude (Fig. 3a). Although the mRNA expression of eNOS was unchanged, the protein expression of eNOS showed an increasing trend with increasing stretch magnitude (Fig. 3b, Table 1). Protein expression of eNOS was significantly higher at 15% stretch magnitude (248 ± 66%) relative to the 0% (100%; P < 0.05) static control (Fig. 3b).

### Pulsatile stretch downregulates phosphorylation of eNOS at S1177 in cerebral ECs

Phosphorylation of eNOS at serine-1177, a common activation site of eNOS, was quantified using western blotting under the same stretch conditions as for eNOS. In contrast to the upregulated eNOS protein expression levels in response to stretch, the phospho-eNOS at serine-1177 was downregulated at 5% (70 ± 11%; P < 0.05), 10% (46 ± 8%; P < 0.0001) and 15% (23 ± 3%; P < 0.0001) stretch magnitudes relative to the 0% static control (100%; Fig. 3c). The phospho-eNOS (peNOS) level at the 5% stretch magnitude (70 ± 11%; P < 0.001) was significantly higher than that at the 15% stretch magnitude (23 ± 3%; Fig. 3c). Linear regression analysis revealed an inversely proportional relationship between peNOS and stretch magnitude (Fig. 2c, Table 1).

### Pulsatile stretch modifies the expression of the inflammatory marker, ICAM-1, in cerebral ECs

**Figure 4:**
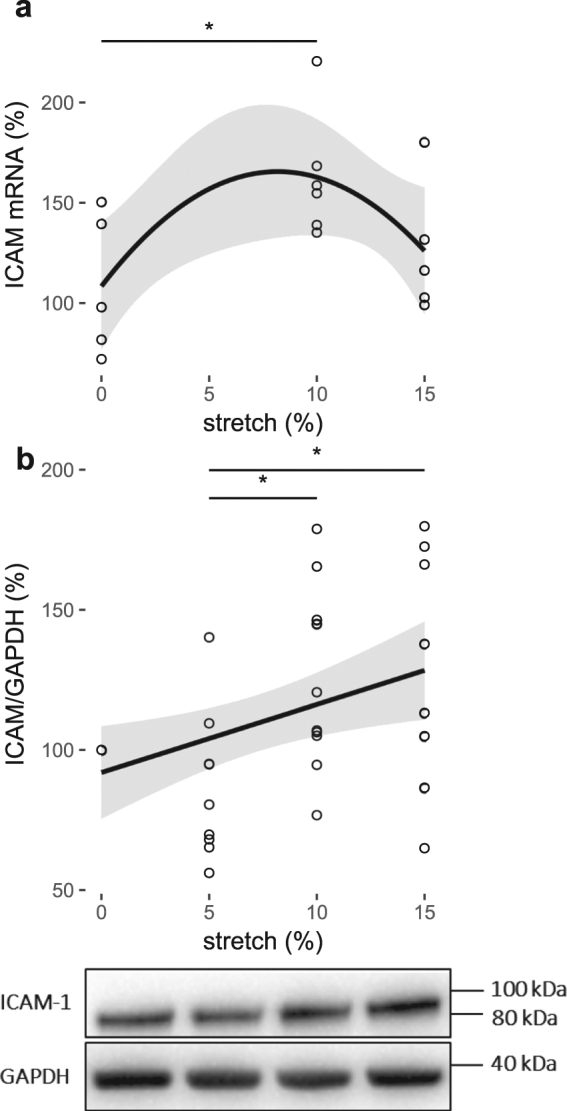
Pulsatile stretch modulates inflammatory marker ICAM-1 expression. % control mRNA expression (**a**) and densitometric analysis of % control band intensity relative to GAPDH and the representative western blots of protein expression (**b**) of ICAM-1 in HCMECs subjected to 18-hour-cyclic (1 Hz) stretch between 0% and 15% magnitude. *P < 0.05. Regression in (**a**), R^2^ = 0.07, P = 0.33. n = 5–10, analyzed using One-way ANOVA with post-hoc Tukey-corrected multiple comparison tests. Solid line shows the regression fit for the data and the shaded region the 95% confidence interval of the regression (regression statistics in Table 1).

ICAM-1 is an inflammatory marker that is regulated by eNOS mediated-NO^13^. ICAM-1 mRNA expression significantly increased at 10% stretch magnitude (163 ± 13%, P < 0.05; Fig. 4a) relative to the static control (108 ± 16%) after 18 hours. Protein expression of ICAM-1 was upregulated at pulsatile stretch magnitudes of 10% (125 ± 10%, P < 0.05) and 15% (128 ± 15%, P < 0.05) compared to the 5% pulsatile stretch condition (87 ± 9%; Fig. 4b). Linear regression of ICAM-1 protein expression showed a similar linear trend to that of APP and eNOS, increasing with increased stretch magnitude (Fig. 4b, Table 1).

### Long-term pulsatile stretch does not significantly affect cell death in cerebral ECs

**Figure 5:**
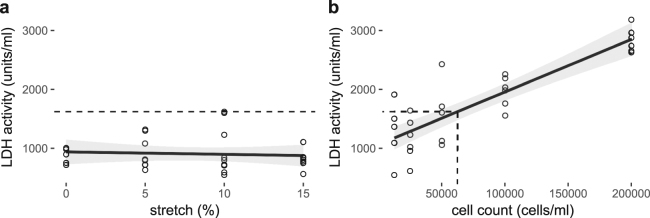
LDH activity relative to the stretch magnitude and corresponding cell death. The dashed line indicates maximum LDH activity observed in experiment conditions (**a**) and the corresponding dead cell count (**b**), a maximum cell death of 8% of the initial cell seeding density in the stretching experiments with the average cell death being much less than that. n = 5–10, analyzed using One-way ANOVA with post-hoc Tukey-corrected multiple comparison tests. Solid line shows the linear regression for the data and the shaded region the 95% confidence interval of the regression (regression statistics in Table 1).

Since upregulation of APP, eNOS and ICAM-1 protein levels and Aβ42 secretion in response to 18 hours of stretch at 0%, 5%, 10% or 15% stretch magnitudes could collectively indicate a stress response, lactose dehydrogenase (LDH) activity, as a marker for stressed and dying cells, was measured after EC’s had been subjected to the same stretch conditions (Fig. 5a). The range of LDH activity measured in the stretching experiments was 296 to 1620 units/ml. There was no increase in LDH activity with increased stretch magnitude (Fig. 5a, Table 1). The highest singular sample dead cell count was approximately 62,000 cells/ml (Fig. 5a,b) calculated based on the linear equation of the standard curve. Accordingly, the maximum overall dead cell count approximated to 8% of the initial cell seeding density in the stretching experiments, with the average cell death being much less than 8% (Fig. 5a,b).

## Discussion

Amyloid deposits, a hallmark of AD, are mainly composed of Aβ aggregates^1^. One of the proposed risk factors for AD is hypertension, which could imply increased pulsatile stretch on microvascular vessel walls, particularly, since pulse pressure and large vessel stiffness increases with age^10,11,14^. Based on this knowledge, the current study firstly evaluated the possible effect of increasing pulsatile stretch magnitude as a mechanistic stimulus on the expression and processing of APP, the precursor for Aβ, and secreted Aβ levels in a cell culture model of cerebral microvascular ECs. Notably, 5% stretch is considered physiological while 15–20% stretch quoted as pathological^15^. In this study, 20% stretch magnitude was omitted due to substantial cell detachment after 18 hours of stretch, so that a maximal possible magnitude of 15%, which yielded a sufficient protein yield with minimal cell detachment was utilised.

Aβ peptides are cleaved fragments of APP, of which in the normal brain, the accumulation is prevented through adequate regulation of APP processing and clearance^1^. However, in AD, Aβ is accumulated and deposited as Aβ plaques, which is considered to occur via several mechanisms including over-expression of APP, enhanced cleavage of APP to Aβ, and reduced clearance of Aβ^1,16^. APP is cleaved and processed by a number of proteolytic enzymes^1^. The formation of Aβ peptides such as Aβ40 and Aβ42, results predominantly from the cleaving of APP by BACE-1 via the amyloidogenic pathway^1^. Current study showed that increasing the magnitude of pulsatile stretch could lead to upregulation of APP expression and amyloidogenic processing enzyme, BACE-1, and Aβ42 secretion (Figs 1 and 2). The expression of APP was regulated at both mRNA and protein levels (Fig. 1a,b), which coincided with increased BACE-1 protein expression levels. The upregulation or activation of BACE-1 was indicative of amyloidogenic processing of APP that leads to increased Aβ levels. The amyloid plaques in AD consist of two major isoforms of Aβ, Aβ42 and Aβ40^17^. The interaction between Aβ42 and Aβ40 has been considered to play a critical role in Aβ deposition^17^. Assessment of Aβ42/Aβ40 ratio has been suggested to be one of the important diagnostic markers for AD pathogenesis^18,19^. To address this, the secreted Aβ40 and 42 levels were investigated. The secretion of Aβ40 did not change significantly although the secretion of Aβ42, which is considered to exhibit higher cellular toxicity compared to other Aβ fragments, proportionally increased with increasing pulsatile stretch (Fig. 2a,b), indicating that Aβ42/Aβ40 ratio was shifted towards Aβ42 (Fig. 2c). Many studies suggested that Aβ42 is the major component of amyloid plaques in AD brains, while Aβ40 is detected only in a subset of plaques^17,20–22^. They explained that the Aβ42 deposition precedes Aβ40 deposition and the initial Aβ42 aggregation does not involve Aβ40 while preferential binding of Aβ42 to extracellular matrix or cell membrane may lead to a high local Aβ42 concentration in the amyloid plaques^17,20–23^. Together, findings of the current study emphasise that increased pulsatile stretch could modulate the expression and processing of APP towards the amyloidogenic pathway, favouring formation of Aβ42.

To the best of our knowledge, the direct effect of pulsatile stretch on the expression and processing of APP, and Aβ secretion in cerebral ECs has not been studied before. However, there is considerable evidence supporting the hypothesis that pulsatile stretch modifies the expression and/or processing of APP^24^. Traumatic brain injury, in a non-transgenic mouse model, where sudden mechanical stress induces axonal damage, has led to upregulated cerebral APP expression and BACE-1^24^. A recent study demonstrated a significant correlation between amplitude of retinal vascular pulsatility and neocortical Aβ scores in an elderly cohort including clinical and pre-clinical AD patients^25^. Additionally, hypertension or increased pulse pressure, both of which could lead to elevated microvascular pulsatility, are associated with increased risk of pre-symptomatic AD in cognitively normal elderly subjects^4,11^. Angiotensin-II induced hypertension in Tg2576 mice, an AD model in which both circulating and cerebral Aβ is elevated due to a mutation in APP, has resulted in increased microvascular amyloid deposition and enhanced BACE-1 mediated-amyloidogenic APP cleavage^12^. Thus, our study substantiates previous observations and suggests that mechanical stretch alters the expression and processing of APP. This adds weight to the hypothesis that increased microvascular pulsatile stretch magnitude, which could be a result of hypertension, or high pulse pressure as is the case in isolated systolic hypertension, may have consequences in terms of cerebral Aβ load. That is, the high pulse pressures generated within the larger vessels in the milieu of hypertension, if transmitted to the microvascular beds as increased microvascular stretch, could act as a mechanical switch for the endothelium to overexpress APP and favour APP cleavage into Aβ. Although the present study showed the direct effect of pulsatile stretch on the expression of APP and the secretion of Aβ, it was not possible to investigate the clearance of Aβ once released into the supernatant. Of note, the assumption here is that the kinetics of physiological Aβ clearance remain unchanged such that Aβ would accumulate over time. The physiological and pathophysiological levels of Aβ42 in human circulation are stated to be 50 pM and 4.3 nM respectively^26^. The range of Aβ42 in this study was 0.1–0.4 pM. Although this concentration fell below the physiological level of circulating Aβ42, it would still substantially account for the overall Aβ42 considering that the physiological endothelial milieu would be much larger in scale to the experimental conditions and if it would accumulate over time. Given that the present study was performed over 18 hours in a small number of cells, and that Aβ accumulates over decades in the pathophysiology of AD across a large vascular bed, the amounts reported in this study would be remarkable if it were to accumulate over time. Moreover, physiologically, there are other sources of Aβ42 that contribute to the circulating Aβ42 pool such as neurons. Notably, Aβ40 and Aβ25–35 at nanomolar to micromolar ranges showed a concentration-dependent effect on EC proliferation, thus even lower concentrations of Aβ may have profound effects on EC function, which are key regulators of the blood-brain barrier that is also important in Aβ clearance^27^.

The expression of APP is altered at different magnitudes of pulsatile stretch (Fig. 1a,b). An inverse correlation between APP expression and eNOS activity has been reported^2,6,8^. Over-expression of APP in Tg2576 mice has been demonstrated to lead to endothelial dysfunction, thereby mitigating endothelial vasorelaxation with a concomitant decrease in bioavailable NO^2^. Austin *et al*.^6,8^ further elaborated a significant role of NO in modulating the expression of APP. In human brain microvascular ECs treated with the NOS inhibitor, N (G)-Nitro-L-Arginine Methyl Ester, and in eNOS^−/−^ mice, it was shown that the expression of APP and BACE-1, and Aβ levels were up-regulated^6^. These effects in eNOS^−/−^ mice were reversed by supplementation with NO by treating the animals with nitroglycerine, which resulted in a marked increase in cyclic guanosine monophosphate (cGMP) levels^8^. An intriguing feedback mechanism between NO bioavailability and Aβ can be illustrated by these studies along with the study of Rajadas *et al*.^28^, which showed that Aβ1–42 treatment caused an upregulation of eNOS expression^28^. This is consistent with a compensatory mechanism dependent on NO bioavailability as increased Aβ may trigger eNOS expression to compensate for the reduced bioavailable NO levels^28^.

The present study substantiates the existing evidence that APP and BACE-1 expression and Aβ secretion proportionally increased with stretch and showed similar effects on eNOS expression and phosphorylated eNOS at serine-1177, a common activation site of eNOS that is upstream of endothelial-dependent NO production. Although the overall protein expression of eNOS (Fig. 3a,b) was increased, the peNOS levels (Fig. 3c) were decreased in response to pulsatile stretch. Previous studies have elaborated that pulsatile stretch could induce peNOS at S1177^29,30^. However, these studies were done within 1 hour of stretch in contrast to 18 hours in the current study. Therefore, the results show that, regardless of the overall increase in eNOS expression, the phosphorylation of eNOS at S1177 is downregulated by long-term pulsatile stretch that could in turn lead to a decrease in endothelial-dependent NO production, indicative of a dysfunctional endothelium. Consistently, Singh *et al*.^31^ also reported that C-reactive protein, a cardiovascular risk marker leading to endothelial dysfunction, causes decreased eNOS activity and NO production by downregulating eNOS phosphorylation in ECs without changing the total eNOS abundance. Of note, the presence of additional bands on total eNOS blots under stretched versus not-stretched conditions further confirmed that pulsatile stretch could also modulate the processing of eNOS (Fig. 3b). However, the mRNA expression of eNOS appeared to be unchanged in response to stretch (Fig. 3a), in contrast to the protein expression (Fig. 3b). Many stimuli such as hypoxia could decrease-eNOS stability, and thus the unchanged-eNOS mRNA levels in our study could be explained by the stability of mRNA being compromised due to stretch^32^. Nonetheless, the overall results suggested that eNOS may be pivotal in regulating the expression and processing of APP, since concomitant fluctuations of APP, Aβ and eNOS protein expression and phosphorylation levels at different levels of stretch indicated a possible compensatory mechanism, consistent with previous findings.

Fluctuations in eNOS expression and phosphorylation due to stretch suggest parallel activation of endothelial inflammatory pathways. ICAM-1 is an inflammatory marker, which can be regulated by NO levels and is also associated with AD-plaques, hence it was also investigated^5,33^. ICAM-1 mRNA was significantly increased at 10% compared to the static control (Fig. 5a), of which the protein levels were upregulated at both 10% and 15% stretch magnitudes compared to the 5% (Fig. 5b). This finding is supported by previous studies that have shown that ICAM-1 expression was upregulated with pulsatile stretch^34^. Increased ICAM-1 expression has been associated with AD-plaques^5^. Thus, concomitant ICAM-1 and APP increase after 18 hours of cyclic stretching in the present study suggests that increased APP is associated with increased ICAM-1 expression.

A recent proteomics study that utilised the same approach as the present study, where human cerebral endothelial cells (HCMECs) were stretched for 18 hours at 1 Hz at 5% or 20% stretch magnitudes, revealed that proteins that are related to inflammation and APP were regulated by stretch^35^. Phospholipase-A2-activating-protein that could mediate Aβ-driven-inflammatory responses was downregulated at 5% stretch relative to 0%, implicating the involvement of physiological stretch as a possible inflammatory suppressor^36^. In contrast, at 20% stretch, upregulation of apolipoprotein B-100 (APOB), junctional-adhesion-molecule-A and ELKS/Rab6-interacting/CAST family member-1, a regulator in activating nuclear-factor-kappa-B (NFκB) that elevate ICAM-1 levels, indicated that prolonged-stretch could induce a pro-inflammatory state in ECs^9,37^. Of note, APOB is known to modulate APP metabolism^38^. At 20% stretch, integrin β-3 (ITGB3), which positively regulates eNOS, increased relative to 0%^27^. ITGB3 is also known to interact with low-density-lipoprotein-related-receptor-1 that facilitates Aβ clearance^39^. cAMP-dependent-protein-kinase-catalytic-subunit-β that regulates cAMP-dependent protein kinase activity, which is known to regulate non-amyloidogenic processing of APP, was significantly down-regulated at 20% stretch compared to 5%^40^. Although the present study did not investigate the underlying molecular mechanisms in detail, this proteomic study substantiates possible mechanisms such as aberrant Aβ clearance and cAMP-dependent APP processing, and integrin- and/or NFκB-mediated-eNOS and ICAM-1 signaling, which could be implicated in eventuating deregulation of APP expression, processing and/or clearance and inflammatory responses as seen in the current study. Additionally, decreased eNOS phosphorylation at serine 1177 further corroborates the inflammatory state of the stretched cells.

It is important to note that there are factors inherent to the mechanical stretch system that are unavoidable, which may account for the variability seen in some of the data, and is a limitation of the current study. As this is a mechanical (pneumatic) system, the stretch chambers may not have been stretched to the exact set magnitudes at all times accounting for some level of error. Additionally, one representative control sample that matched the cell passage number closest to all stretch levels out of the 5%, 10% or 15% stretch magnitudes was randomly selected to represent the static control for each 5%, 10% and 15% set that was run on a gel at a time. This was to be able to compare between the stretch magnitudes, in which case, all samples had to be run simultaneously on the same gel next to each other. This could introduce a some level of variability as there are variations between controls from batch to batch. Thus, the above factors could introduce variability within the data. However, importantly, despite the variability introduced by these factors, the recorded effects are statistically significant, and tighter control of these variables or longer time periods of stretching would likely increase the confidence in these significant differences.

Future studies are warranted to further investigate whether the associations between APP, eNOS and ICAM-1 in response to pulsatile stretch were directly interdependent. Taken together, the results can be implicated in expanding the existing knowledge on the effect of microvascular pulsatility as a mechanical stimulus that modulates endothelial APP, eNOS and ICAM-1 in the context of AD. Modulation of pulsatility of blood microvessels may pose beneficial effects in alleviating the potential deleterious effects on AD progression.

## Materials and Methods

### Cell culture and maintenance

Immortalized human cerebral microvascular endothelial cells-SV40 (HCMEC-SV40) were purchased from Applied Biological Materials Inc. and maintained in M199 media (Sigma-Aldrich) supplemented with 10% fetal bovine serum and 1% penicillin/streptomycin at 37 °C with an atmospheric humidity of 5% CO_2_.

Cells were seeded on fibronectin-coated (375 μg/ml) silicon chambers 24-hours prior to stretching at a seeding density of 8 × 10^5^ cells/ml. Cells were stained with trypan blue and counted using the Countess automated cell counter (Life Technologies) according to the manufacturer’s protocol. Passages 17–19 were used for all experiments.

### Cyclic stretching of ECs

Cells were subjected to uni-axial pulsatile stretch as previously described^41^, using the ShellPa stretch system (B-Bridge International) under culture conditions mentioned above. The silicon chambers have 200 μm thick transparent bottoms with side-wall thickness of 400 μm to support uniform stretching across the cell substrate. The chambers were mounted in the stretching apparatus where one end of the chamber is fixed and the other connected to an actuator operated by compressed air. The actuated arm stretched the chambers at a rate of 1 Hz for 18 hours in all experiments at a maximum extension of either 5, 10 or 15%. Control condition consisted of the same placement of the chambers, but with no cyclic extension applied for the 18-hour period.1 Hz was chosen based on previous stretch studies^41–43^ and is in the range of resting human heart rate. The degree of stretch (%) is defined the as relative elongation of the chamber.

### Gene expression quantification using real-time quantitative reverse transcription polymerase chain reaction (RT-qPCR)

Total RNA was extracted using TRIZOL (Invitrogen) according to the manufacturer’s instructions after the 18-hour period and was normalized to 100 ng/μL using the Nano-Drop spectrophotometer. A 260/280 ratio of approximately 1.8–2.0 was used to ensure the RNA quality. Total RNA of 0.4 μg was then reverse transcribed using the SuperScript VILO complementary deoxyribonucleic acid (cDNA) synthesis kit (Life Technologies) according to the manufacturer’s protocol. Negative control samples (RT-) were prepared by substituting the reverse transcriptase with dH_2_O as a control for genomic contamination. This was followed by real-time qPCR using TaqMan® gene expression assays and TaqMan® gene expression master mix (Applied Biosystems) according to the manufacturer’s protocol. The qPCR program comprised 120 s at 50 °C (incubation for optimal activity of uracil-DNA glycosylase), 600 s at 95 °C (optimal AmpliTaq Gold, Ultra-Pure enzyme activity), 15 s at 95 °C (denaturation) followed by 60 s at 60 °C (annealing and extension) repeated for 40 cycles.

The following pre-designed primer-probe TaqMan® assays by Life Technologies: APP (Hs00169098_m1); eNOS (Hs01574659_m1); and β2 microglobulin (Hs00984230_m1). Relative changes in mRNA levels were determined by the comparative ΔΔCT method normalised with human β2 microglobulin^44^.

### Protein expression quantification using Western blotting

After 18 hours of stretching, the media was aspirated and the cells were briefly washed twice with cold phosphate-buffered-saline followed by addition of RIPA buffer (50 mM Tris-HCl, pH 7.4, 150 mM NaCl, 5 mM EDTA, 10 mM NaF, 10 mM sodium pyrophosphate, 1% IGEPAL CA-630, 0.5% sodium deoxycholate, 0.1% sodium dodecyl sulfate (SDS)) with protease inhibitor (Sigma-Aldrich) added immediately prior to lysis. Cells were then harvested by scraping and the lysates were centrifuged at 14,000 × g for 15 minutes at 4 °C after sonication. The protein content was quantified using BCA assay (Pierce) according to the manufacturer’s protocol.

40 µg of cell lysates were mixed with 4 × NuPAGE sample buffer (Life Technologies) and were heated at 44 °C for 10 minutes before loading on 10% or 4–12% NuPAGE® Novex® Bis-Tris gels. The polyvinylidene difluoride (PVDF) or nitrocellulose membranes were blocked in 5% skim milk buffer in TBS-T (Tris buffered saline-Tween; 20 mM Tris-HCl, pH 7.4, 0.5 M NaCl, 0.1% Tween 20) to be probed with anti-APP (1:1000; Covance), anti-eNOS (1: 250; Cell Signaling Technologies), anti-peNOS at S1177 (1:1000; Cell Signaling Technologies), anti-BACE-1 (1:500; Cell Signaling Technologies), anti-ICAM-1 (1:1000; R&D) or anti-GAPDH (1:1000) antibodies followed by secondary antibody incubations (1:2000; R&D) as previously described^45^. The blots were developed using Clarity™ Western ECL Substrate Kit (Bio-Rad) according to the manufacturers’ protocol. The protein bands were quantified using the Image Lab 5.1 software within the linear range of detection as relative % control normalized to GAPDH (Bio-Rad Labs. Inc). Cropped blots are presented in Figures with the uncropped blot provided in the Supplementary material.

### Measuring secreted Aβ42 using ELISA

The supernatants were collected after 18 hours of stretch and were analysed for secreted Aβ42 according to the manufacturer’s protocol using a commercial ELISA kit (KHB3544, Life Technologies). The absorbance was read at 450 nm and the Aβ42 levels were calculated using a standard curve in pg/ml.

### Measurement of lactose dehydrogenase (LDH) Activity

HCMECs were seeded on a 96-well plate at seeding densities of 2, 1, 0.5, 0.25 and 0.125 × 10^5^ cells/ml. Cells were then treated with 2 mM H_2_O_2_ (tested toxic to HCMECs) for 24 hours and the supernatants were collected for LDH assay (MAK066, Sigma). According to the manufacturers’ protocol, a standard curve of [NADH] versus absorbance read at 450 nm was generated to calculate the LDH activity in units/ml. A standard curve of number of H_2_O_2_-treated cells (taken as dead cell count) versus the corresponding LDH activity was generated to determine the correlation of dying cells with measured LDH activity at different pulsatile stretch magnitudes.

### Statistical data analysis

Data was collected in 5–10 replicates from individual experiments for each condition. Results were analyzed using One-way ANOVA with post-hoc Tukey-corrected multiple comparison tests, or linear regression analysis, using GraphPad PRISM version 6.05.

## Electronic supplementary material

Supplementary material

## References

[1] Ben Halima S (2016). Specific Inhibition of β-Secretase Processing of the Alzheimer Disease Amyloid Precursor Protein. Cell Rep..

[2] d’Uscio LV (2012). Activation of PPARδ prevents endothelial dysfunction induced by overexpression of amyloid-β precursor protein. Cardiovasc. Res..

[3] Brickman AM (2015). Cerebral autoregulation, beta amyloid, and white matter hyperintensities are interrelated. Neurosci. Lett..

[4] Nation DA (2013). Pulse pressure is associated with Alzheimer biomarkers in cognitively normal older adults. Neurology.

[5] Apelt J, Leßig J, Schliebs R (2002). β-amyloid-associated expression of intercellular adhesion molecule-1 in brain cortical tissue of transgenic Tg2576 mice. Neurosci. Lett..

[6] Austin SA, Santhanam AV, Katusic ZS (2010). Endothelial nitric oxide modulates expression and processing of amyloid precursor protein. Circ. Res..

[7] Lamoke F (2015). Amyloid β peptide-induced inhibition of endothelial nitric oxide production involves oxidative stress-mediated constitutive eNOS/HSP90 interaction and disruption of agonist-mediated Akt activation. J. Neuroinflammation.

[8] Austin SA, D’Uscio LV, Katusic ZS (2013). Supplementation of nitric oxide attenuates AβPP and BACE1 protein in cerebral microcirculation of eNOS-deficient mice. J. Alzheimers Dis..

[9] Tan Y (2014). Stiffening-induced high pulsatility flow activates endothelial inflammation via a TLR2/NF-κB pathway. PLoS One.

[10] Kurata T (2015). Long-term effect of telmisartan on Alzheimer’s amyloid genesis in SHR-SR after tMCAO. Transl. Stroke Res..

[11] Langbaum JBS (2012). Blood pressure is associated with higher brain amyloid burden and lower glucose metabolism in healthy late middle-age persons. Neurobiol. Aging.

[12] Faraco G (2016). Hypertension enhances Aβ-induced neurovascular dysfunction, promotes β-secretase activity, and leads to amyloidogenic processing of APP. J. Cereb. Blood Flow Metab..

[13] Xu S, Zhou X, Yuan D, Xu Y, He P (2013). Caveolin-1 scaffolding domain promotes leukocyte adhesion by reduced basal endothelial nitric oxide-mediated ICAM-1 phosphorylation in rat mesenteric venules. Am. J. Physiol. Heart Circ. Physiol..

[14] Nichols WW (1985). Effects of age on ventricular-vascular coupling. Am J Cardiol.

[15] Gao J (2014). Preconditioning effects of physiological cyclic stretch on pathologically mechanical stretch-induced alveolar epithelial cell apoptosis and barrier dysfunction. Biochem. Biophys. Res. Commun..

[16] Pflanzner T (2011). LRP1 mediates bidirectional transcytosis of amyloid-β across the blood-brain barrier. Neurobiol. Aging.

[17] Gu L, Tran J, Jiang L, Guo Z (2016). A new structural model of Alzheimer’s Aβ42 fibrils based on electron paramagnetic resonance data and Rosetta modeling. J. Struct. Biol..

[18] Kim, J. *et al*. Aβ40 inhibits amyloid deposition *in vivo*. *J. Neurosci*. **27** (2007). 10.1523/JNEUROSCI.4849-06.2007 PMC6672801 17234594

[19] Kuperstein I (2010). Neurotoxicity of Alzheimer’s disease Aβ peptides is induced by small changes in the Aβ42 to Aβ40 ratio. EMBO J..

[20] Takeshi I (1994). Visualization of Aβ42(43) and Aβ40 in senile plaques with end-specific Aβ monoclonals: Evidence that an initially deposited species is Aβ42(43). Neuron.

[21] Fagan AM (2006). Inverse relation between *in vivo* amyloid imaging load and cerebrospinal fluid Aβ42 in humans. Ann. Neurol..

[22] Gravina SA (1995). Amyloid β protein (Aβ) in Alzheimer’s disease brain. Biochemical and immunocytochemical analysis with antibodies specific for forms ending at Aβ40 or Aβ42 42(43). J. Biol. Chem..

[23] Iwatsubo T, Mann DMA, Odaka A, Suzuki N, Ihara Y (1995). Amyloid β protein (Aβ) deposition: Aβ42(43) precedes Aβ40 in down Syndrome. Ann. Neurol..

[24] Loane DJ (2009). Amyloid precursor protein secretases as therapeutic targets for traumatic brain injury. Nat. Med..

[25] Mojtaba Golzan, S. *et al*. Retinal vascular and structural changes are associated with amyloid burden in the elderly: ophthalmic biomarkers of preclinical Alzheimer’s disease. 10.1186/s13195-017-0239-9. 10.1186/s13195-017-0239-9 PMC5335799 28253913

[26] Deane R (2003). RAGE mediates amyloid-β peptide transport across the blood-brain barrier and accumulation in brain. Nat. Med..

[27] Cantara S (2004). Physiological levels of amyloid peptides stimulate the angiogenic response through FGF-2. FASEB J..

[28] Rajadas J (2013). Enhanced Aβ1–40 production in endothelial cells stimulated with fibrillar Aβ1–42. PLoS One.

[29] Kuebler WM (2003). Stretch activates nitric oxide production in pulmonary vascular endothelial cells *in situ*. Am. J. Respir. Crit. Care Med..

[30] Takeda H (2006). Bi-phasic activation of eNOS in response to uni-axial cyclic stretch is mediated by differential mechanisms in BAECs. Life Sci..

[31] Singh U, Devaraj S, Vasquez-Vivar J, Jialal I (2007). C-reactive protein decreases endothelial nitric oxide synthase activity via uncoupling. J. Mol. Cell. Cardiol..

[32] Ho JJD (2013). Active Stabilization of Human Endothelial Nitric Oxide Synthase mRNA by hnRNP E1 Protects against Antisense RNA and MicroRNAs. Mol. Cell. Biol..

[33] Buras, J. A., Stahl, G. L., Svoboda, K. K. H. & Reenstra, W. R. Hyperbaric oxygen downregulates ICAM-1 expression induced by hypoxia and hypoglycemia: the role of NOS. *Am. J. Physiol. - Cell Physiol*. **278** (2000). 10.1152/ajpcell.2000.278.2.C292 10666024

[34] Tian Y, Gawlak G, O’Donnell JJ, Mambetsariev I, Birukova AA (2016). Modulation of Endothelial Inflammation by Low and High Magnitude Cyclic Stretch. PLoS One.

[35] Farhana N, Mohamedali A, Ahn BS, Avolio A, Baker MS (2017). Effects of acute and chronic biomechanical strain on human cerebral endothelial cells in altering their proteome profile. Curr. Proteomics.

[36] Paris D (2000). Soluble β-amyloid peptides mediate vasoactivity via activation of a pro-inflammatory pathway. Neurobiol. Aging.

[37] Schmitt, M. M. N. *et al*. Endothelial Junctional Adhesion Molecule-A Guides Monocytes Into Flow-Dependent Predilection Sites of AtherosclerosisClinical Perspective. *Circulation* **129** (2014). 10.1161/CIRCULATIONAHA.113.004149 24065611

[38] Bjelik A (2006). Human apoB overexpression and a high-cholesterol diet differently modify the brain APP metabolism in the transgenic mouse model of atherosclerosis. Neurochem. Int..

[39] Akkawi S, Nassar T, Tarshis M, Cines DB, Higazi AA-R (2006). LRP and vbeta3 mediate tPA activation of smooth muscle cells. AJP Hear. Circ. Physiol..

[40] He T, Santhanam AVR, Lu T, d’Uscio LV, Katusic ZS (2017). Role of prostacyclin signaling in endothelial production of soluble amyloid precursor protein-α in cerebral microvessels. J. Cereb. Blood Flow Metab..

[41] Naruse K, Yamada T, Sokabe M (1998). Involvement of SA channels in orienting response of cultured endothelial cells to cyclic stretch. Am. J. Physiol..

[42] Naruse K, Sai X, Yokoyama N, Sokabe M (1998). Uni-axial cyclic stretch induces c-src activation and translocation in human endothelial cells via SA channel activation. FEBS Lett..

[43] Suzuki M (1997). Up-regulation of integrin β3 expression by cyclic stretch in human umbilical endothelial cells. Biochem. Biophys. Res. Commun..

[44] Schmittgen TD, Livak KJ (2008). Analyzing real-time PCR data by the comparative CT method. Nat. Protoc..

[45] Wan X-Z (2012). Activation of NMDA receptors upregulates a disintegrin and metalloproteinase 10 via a Wnt/MAPK signaling pathway. J. Neurosci..

