# Supplementary material for: Pulsatile stretch as a novel modulator of amyloid precursor protein processing and associated inflammatory markers in human cerebral endothelial cells
Source: Sci Rep. 2018 Jan 26;8:1689. doi: 10.1038/s41598-018-20117-6 (PMC5786097; doi:10.1038/s41598-018-20117-6)
Supplement: Supplementary file 1 — Supplementary material [file 41598_2018_20117_MOESM1_ESM.pdf]

Pulsatile stretch as a novel modulator of amyloid precursor protein processing and associated inflammatory markers in human cerebral endothelial cells

Supplementary material

Sumudu V. S. Gangoda[1], Bhargava Avadhanam[1], Nurul F. Jufri[2], Eun Hwa Sohn[3], Mark Butlin[1,*], Vivek Gupta[4], Roger Chung[1], and Alberto P Avolio[1]

[1]Department of Biomedical Sciences, Faculty of Medicine and Health Sciences, Macquarie University, Sydney, Australia.
[2]Programme of Biomedical Science, Faculty of Health Sciences, Universiti Kebangsaan Malaysia, 50300 Kuala Lumpur, Malaysia.
[3]Department of Herbal Medicine Resources, Kangwon National University, Samcheok 25949, Republic of Korea.
[4]Department of Clinical Medicine, Faculty of Medicine and Health Sciences, Macquarie University, Sydney, Australia.
[*]corresponding author mark.butlin@mq.edu.au

This supplementary section provides the uncropped version of western blots provided in the main paper figures, as outlined in Table 1.

Table 1. Corresponding uncropped blot supplementary figures of figures in main article.

main article figure	corresponding supplementary figure
Figure 1(b)	Figure S1
Figure 1(c)	Figure S2
Figure 3(b)	Figure S3
Figure 3(c)	Figure S4
Figure 4(b)	Figure S5

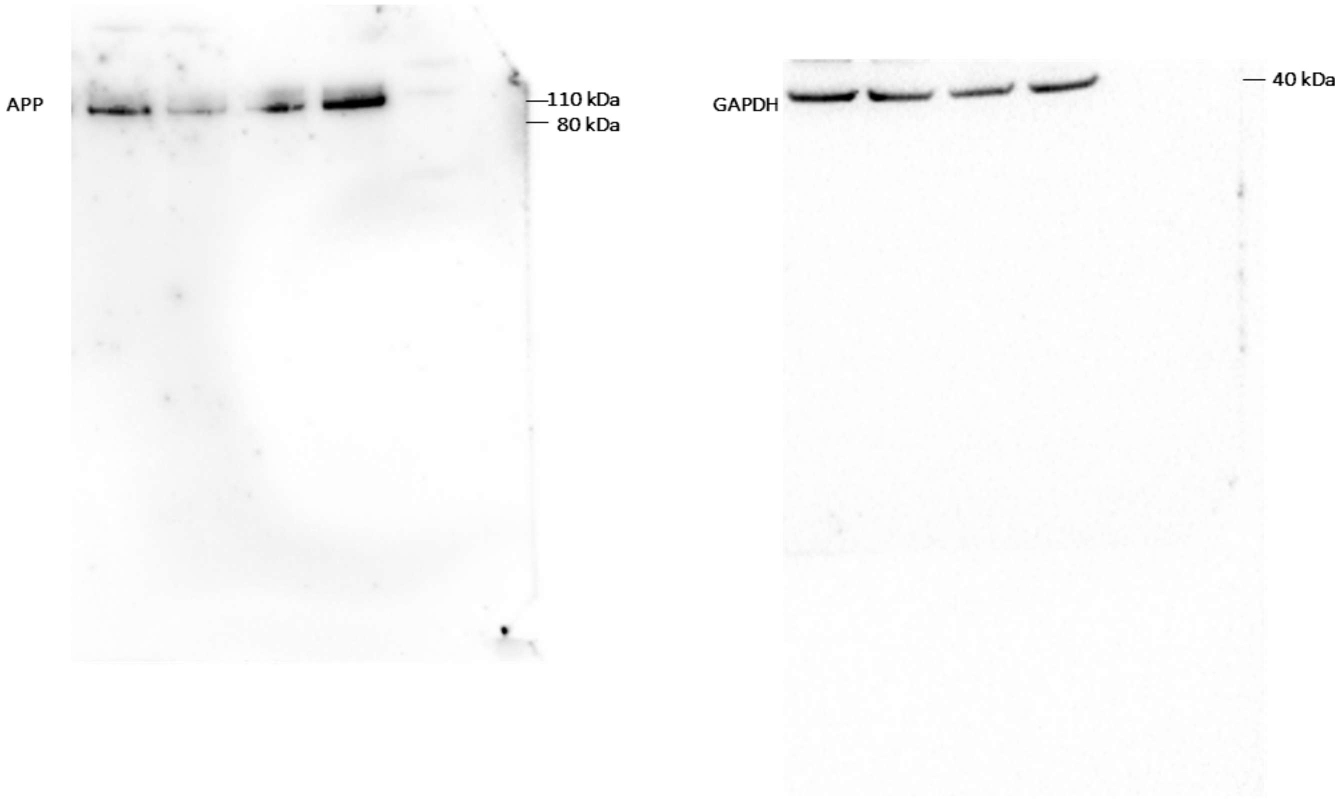

Figure S1. Representative western blot of protein expression of APP expression indicating amyloidogenic processing of APP at different magnitudes of pulsatile (1 Hz) stretch of HCMECs over 18 hours. Uncropped version of Figure 1(b).

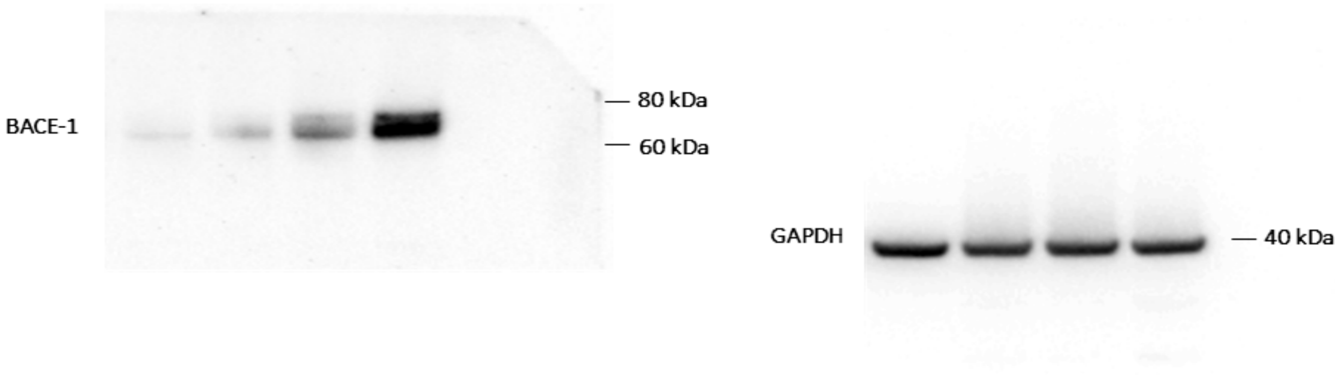

Figure S2. Representative western blot of protein expression of BACE-1 expression indicating amyloidogenic processing of APP at different magnitudes of pulsatile (1 Hz) stretch of HCMECs over 18 hours. Uncropped version of Figure 1(c).

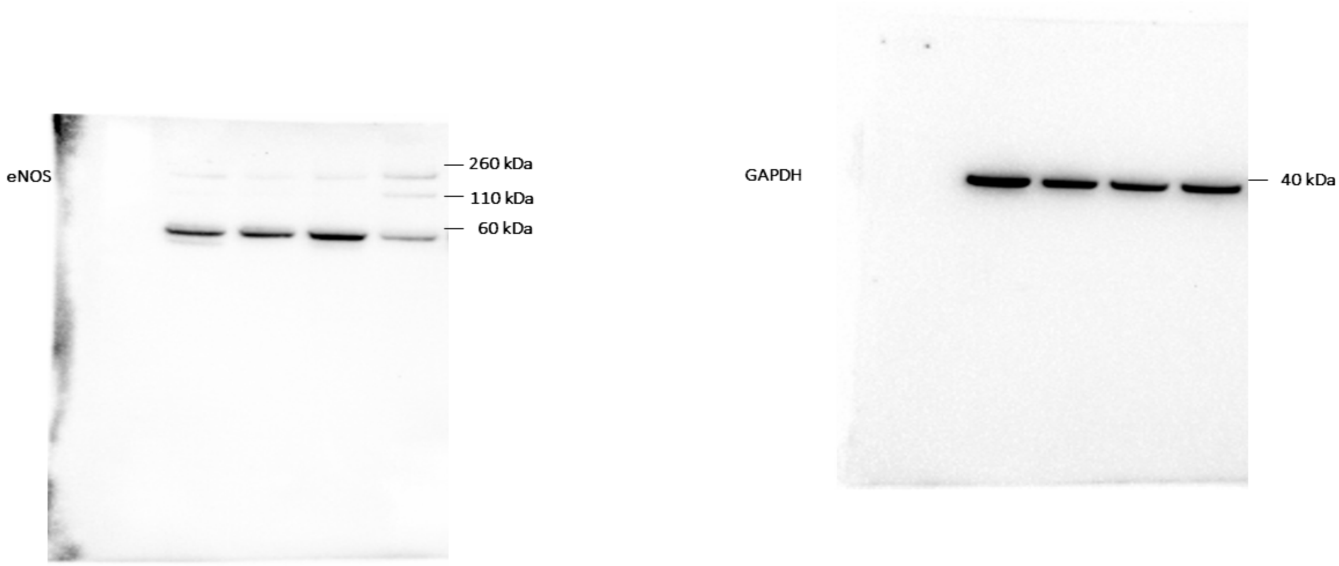

Figure S3. Representative western blot of protein expression of eNOS in response to pulsatile (1 Hz) stretch of HCMECs over 18 hours. Uncropped version of Figure 3(b).

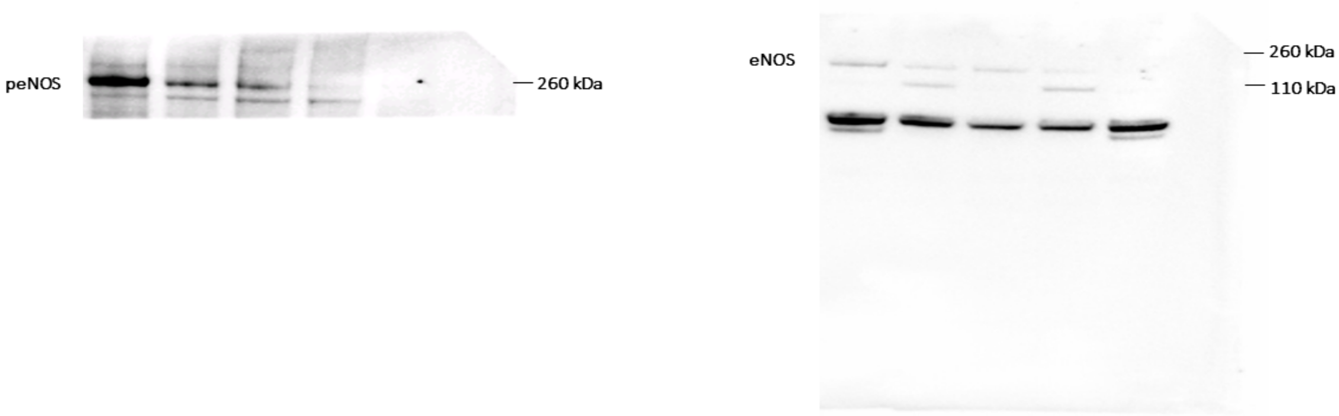

Figure S4. Representative western blots of protein phosphorylation at S1177 of eNOS in response to pulsatile (1 Hz) stretch of HCMECs over 18 hours. Uncropped version of Figure 3(c).

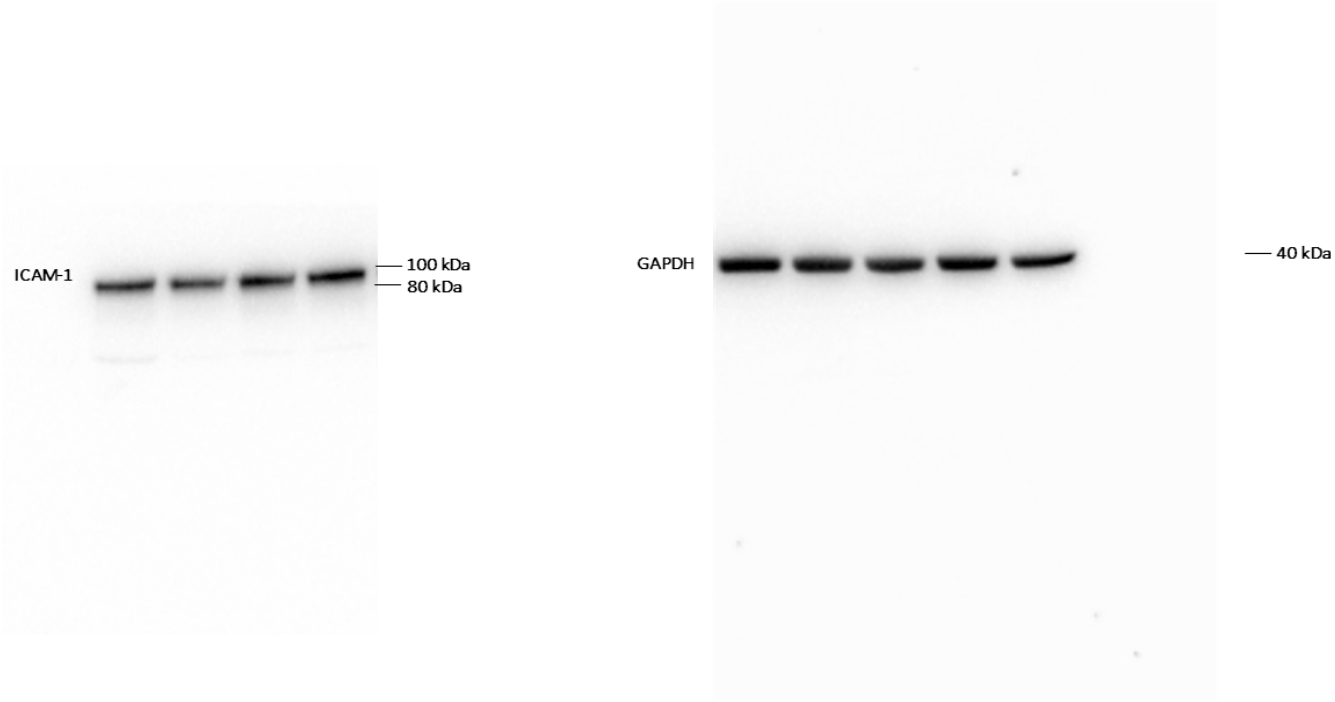

Figure S5. Representative western blots of protein expression (b) of ICAM-1 in response to pulsatile (1 Hz) stretch of HCMECs over 18 hours. Uncropped version of Figure 4(b).
